# *Mesomycoplasma ovipneumoniae* from goats with respiratory infection: pathogenic characteristics, population structure, and genomic features

**DOI:** 10.1186/s12866-023-02964-0

**Published:** 2023-08-14

**Authors:** Chunxia Ma, Ming Li, Hao Peng, Meiyi Lan, Li Tao, Changting Li, Cuilan Wu, Huili Bai, Yawen Zhong, Shuhong Zhong, Ruofu Qin, Fengsheng Li, Jun Li, Jiakang He

**Affiliations:** 1 College of Animal Science and Technology, Guangxi University, Nanning, 530004 Guangxi China; 2 Guangxi Key Laboratory of Veterinary Biotechnology, Guangxi Veterinary Research Institute, Nanning, 530001 Guangxi China; 3 Key Laboratory of China (Guangxi)-ASEAN Cross-Border Animal Disease Prevention and Control, Nanning, 530001 Guangxi China; 4 Guangxi Key Laboratory of Aquatic Genetic Breeding and Healthy Aquaculture, Guangxi Institute of Fisheries, Nanning, 530021 Guangxi China

**Keywords:** *Mycoplasma ovipneumoniae*, Whole-genome sequencing, Pathogenesis, Pan-genome

## Abstract

**Background:**

*Mycoplasma ovipneumoniae* is a critical pathogen that causes respiratory diseases that threaten *Caprini* health and cause economic damage. A genome-wide study of *M. ovipneumoniae* will help understand the pathogenic characteristics of this microorganism.

**Results:**

Toxicological pathology and whole-genome sequencing of nine *M. ovipneumoniae* strains isolated from goats were performed using an epidemiological survey. These strains exhibited anterior ventral lung consolidation, typical of bronchopneumonia in goats. Average nucleotide identity and phylogenetic analysis based on whole-genome sequences showed that all *M. ovipneumoniae* strains clustered into two clades, largely in accordance with their geographical origins. The pan-genome of the 23 *M. ovipneumoniae* strains contained 5,596 genes, including 385 core, 210 soft core, and 5,001 accessory genes. Among these genes, two protein-coding genes were annotated as cilium adhesion and eight as paralog surface adhesins when annotated to VFDB, and no antibiotic resistance-related genes were predicted. Additionally, 23 strains carried glucosidase-related genes (*ycjT* and *group_1595*) and glucosidase-related genes (*atpD_2*), indicating that *M. ovipneumoniae* possesses a wide range of glycoside hydrolase activities.

**Conclusions:**

The population structure and genomic features identified in this study will facilitate further investigations into the pathogenesis of *M. ovipneumoniae* and lay the foundation for the development of preventive and therapeutic methods.

**Supplementary Information:**

The online version contains supplementary material available at 10.1186/s12866-023-02964-0.

## Background

*Mycoplasma ovipneumoniae* is an etiological microorganism of acute or chronic pneumonia in *Caprini*, affecting healthy animals and causing economic damage. Infection outbreaks usually occur in sheep and goat populations and are strongly associated with the transfer of sick individuals to susceptible herds [1, 2]. Domestic sheep and goats serve as asymptomatic carriers of *M. ovipneumoniae* and spread this pathogen to native bighorn sheep populations through close contact [3, 4]. Additionally, epizootic introduction events in sheep and goat populations frequently result in annual pneumonia outbreaks in susceptible juveniles, high flock mortality, and years of reduced herd growth rate [5, 6]. Secretions from individuals with pneumonia or pathogen carriers mainly contribute to the spread of the disease through nasal inhalation of infected droplets [7, 8]. Once infected with *M. ovipneumoniae*, sheep and goats develop severe diseases, including contagious pleuropneumonia, bronchopneumonia, polymicrobial pneumonia, arthritis, keratoconjunctivitis, consolidated lung lesions, and pulmonary abscesses.

Currently, there are few global surveillance records for *M. ovipneumoniae* and some gaps in basic research have resulted from nutritionally limited conditions for in vitro growth (24–72 h with a peak at 48 h), a spherical morphology (pear, flask-shaped cells, and filaments), and a lack of fried egg-like morphology on agar plates [9, 10]. After isolation, microbial identification mainly depends on additional tests such as biochemical testing, PCR, Sanger sequencing, serological testing, or matrix-assisted laser desorption/ionization-time of flight (MALDI-TOF) [1, 11–13]. Direct molecular methods with high resolution for detection using sheep clinical samples without pre-culture, such as real-time quantitative PCR, loop-mediated isothermal amplification (LAMP), and droplet digital PCR, seem promising but lack broad validation [14, 15]. In addition, owing to difficulties in counting *M. ovipneumoniae* cells, antimicrobial susceptibility testing (AST) has been poorly studied; thus, methodological standards are lacking and there are currently no clinical interpretation criteria available [16, 17]. Whole genome sequencing (WGS) is widely used to characterize microbial genomes. Theoretically, WGS-based strategies allow the classification of housekeeping genes, epidemiological relationships, serology, virulence genotypes, and antimicrobial resistance genotypes of all classes of infectious agents [18, 19]. A recent survey of *Enterococcus spp.* isolated from different environments confirmed that traditional molecular techniques are less effective than WGS for annotating antimicrobial resistance genes (ARGs) and virulence genes [20]. However, this strategy faces four critical challenges: time consumption, data analysis, reporting interpretation, and cost. Thus, WGS should be conceptually and technologically optimized to its greatest potential.

Therefore, this study aimed to isolate and identify *Mycoplasma spp.* circulating in goats with respiratory infections in Guangxi, China, using WGS to study the population structure, ARGs, and virulence genes of this microorganism. We aimed to enrich genomic resources and provide fundamental genomic insights to facilitate molecular diagnostics and pathogenic microbial therapies.

## Results

### Isolation and pathogenic characteristics of *M. ovipneumoniae* strains

**Fig. 1:**
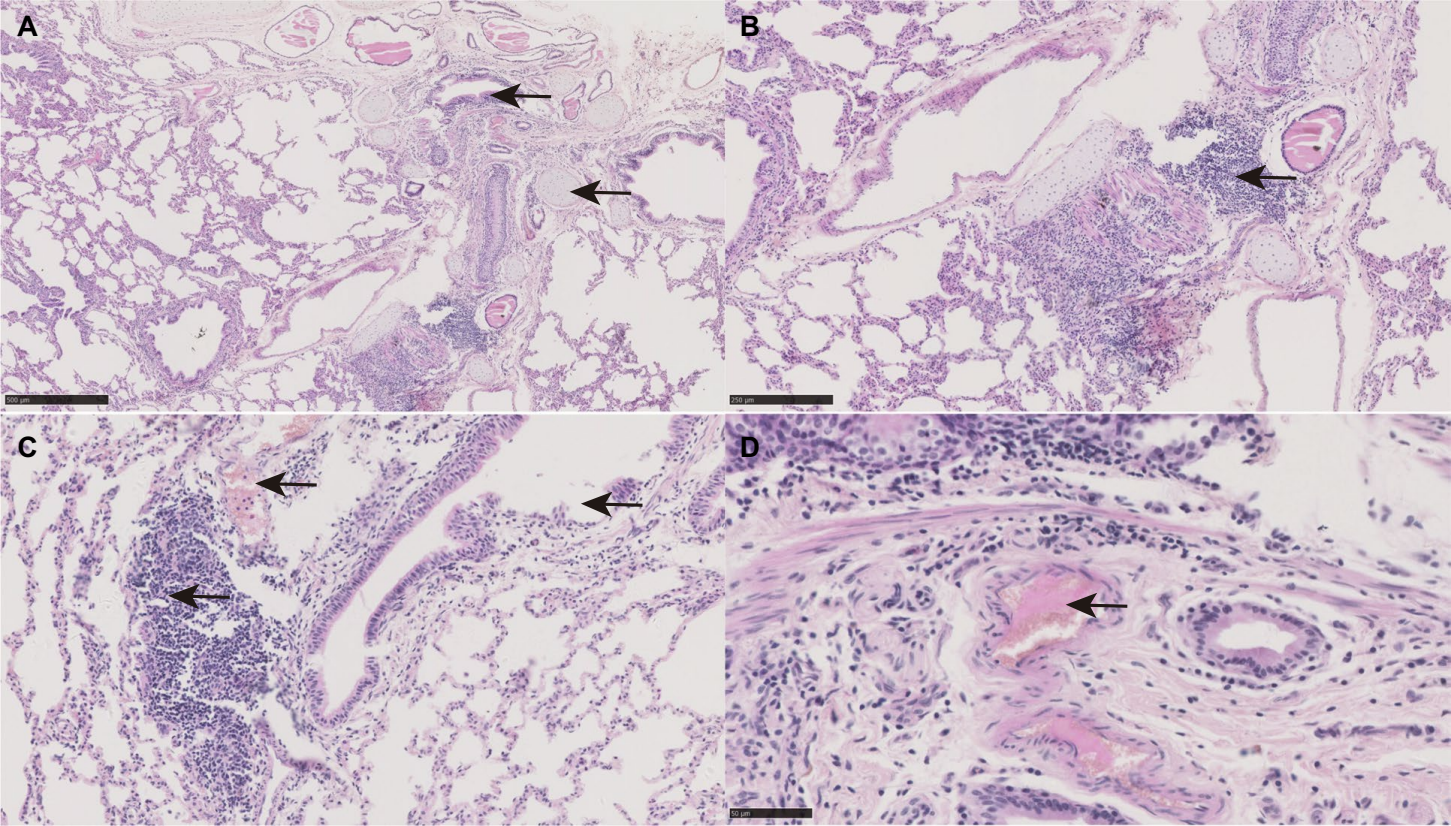
Morphology of goat lung tissue. **A** and **B** represent cell morphology of goat lung tissue after *Mycoplasma ovipneumoniae* NN-MO infection. **C** and **D** represent morphology of goat lung tissue after *M. ovipneumoniae* BHF6 infection

Before pathogen isolation, we performed an epidemiological analysis of the goat population in Guangxi; 87 cases revealed typical clinical symptoms, including respiratory (83.9%), diarrhea (9.2%), and parasitic symptoms (6.8%). Further exploration of the purified microorganisms identified 49.6% as *M. ovipneumoniae*, 12.4% as a subspecies of *Mycoplasma filiformis*, 12.4% as *Manniella hemolyticus*, 13.9% as *Escherichia coli*, 5.8% as *Klebsiella pneumoniae,* and 5.8% as *Cryptobacterium pyogenes* according to the 16S rRNA sequences. Goats infected with *M. ovipneumoniae* had light-yellow lungs that produced emphysema, diffuse congestion, reddish-brown areas, edema, and hemorrhage. Simultaneously, pulmonary bullae (emphysema) were visible because of alveolar fusion (Fig. 1A). In addition, swollen mucosa, congestive blood vessels in the mucosal and submucosal layers, inflammatory cell infiltration, and degeneration and necrosis of the mucosal epithelial cells were observed (Fig. 1B and C). Moreover, lymphatic vessel dilation, lymphatic thrombosis, interstitial capillary thrombosis, and pulmonary interstitial necrosis were also detected (Fig. 1D).

*M. ovipneumoniae* strains NN–MO, MSM, and YZM were obtained from the lung tissue, and strains BHF6, DH1, DH4, FS2, GT1, and GXHX211028 were isolated from nasal swabs. Single colonies of *M. ovipneumoniae* and *M. filiformis* appeared as tiny dew drops (Figure S1 A) and fried eggs (Figure S1 B) on the agar plate where the raised center was wider in some colonies of *M. ovipneumoniae* and smaller in a few colonies upon light microscopic examination (Figure S1 C). All isolates were identified as *M. ovipneumoniae* by Sanger sequencing, using specific primers. We further used the strains BHF6 and FS2 to infect goats and found that the infected goats exhibited lung consolidation typical of bronchopneumonia, as shown in Fig. 1. Microscopic examination of the lesions revealed tracheal and bronchial mucosal edema. The cilia and microvilli were identified. Hyperemia, inflammatory cell infiltration, mitochondrial swelling, and cristae were also observed. In addition, the local vascular endothelial membranes were damaged.

### Genomic statistics of *M. ovipneumoniae* strains

Relevant information regarding the whole-genome sequences of the nine *M. ovipneumoniae* strains is presented in Table 1. The N50 values of the nine assembled genomes ranged from 46.745 to 471.097 kb. Eighty scaffolds were used for each assembly. Moreover, the GC content of all strains ranged from 28.74% to 28.96%, which is in accordance with the *M. ovipneumoniae* genomes available in the NCBI database (Table S1). In addition, the coding genes in each strain accounted for more than 1,425 genes, whereas the number of non-coding genes was less than 41.

**Table 1:** Genomic statistics of 9 *Mycoplasma ovipneumoniae* genomes sequenced in this study

Strain	Number of sequences	Total length (bp)	CG content (%)	Average lengths	Average length (kb)	N50	N50 (kb)	Coding gene	Non-coding gene
131213NN-MO	43	1,024,299	28.9	23,820.91	23.82	46745	46.745	1447	33
BHF6	9	996,893	28.95	110,765.89	110.77	197056	197.056	1450	35
DH1	19	1,053,909	28.78	55,468.89	55.47	109416	109.416	1532	34
DH4	8	992,882	28.96	124,110.25	124.11	187061	187.061	1429	34
FS2	7	994,476	28.95	142,068	142.07	245050	245.05	1425	34
GT20201111	23	1,053,882	28.74	45,820.96	45.82	87661	87.661	1531	34
GXHX211028	79	1,640,719	28.91	20,768.59	20.77	471097	471.097	2296	40
MSM	35	1,034,546	28.95	29,558.46	29.56	82198	82.198	1473	34
YZM	38	1,131,018	28.79	29,763.63	29.76	65233	65.233	1648	34

**Fig. 2:**
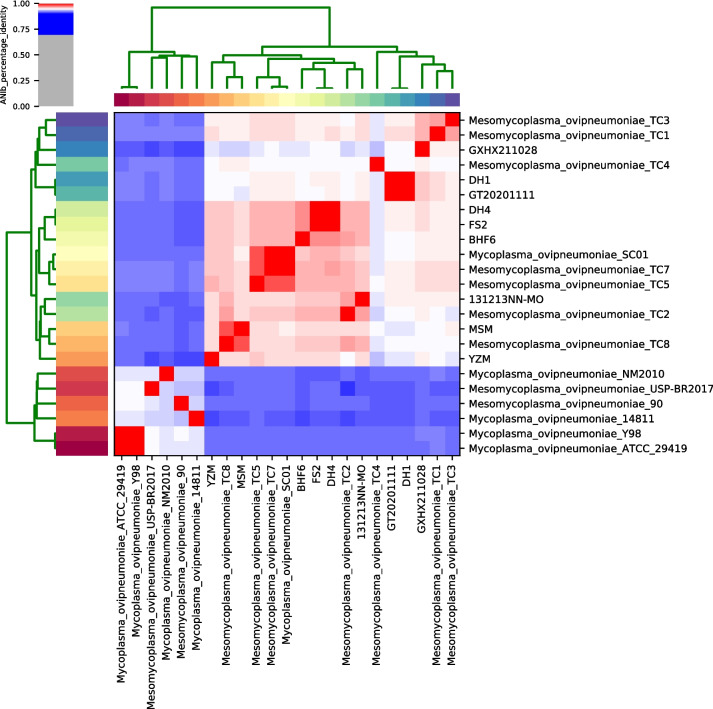
Hierarchical clustering in two dimensions of pairwise average nucleotide identity (ANI) of 23 *Mycoplasma ovipneumoniae* strains. Pairwise ANI values are presented as a heatmap

Pairwise average nucleotide identity (ANI) values of the nine strains and fourteen publicly available *M. ovipneumoniae* genome sequences were analyzed. Two clades of microbial strains were identified in the dendrogram constructed by clustering pairwise ANI values (Fig. 2). Closely related strains from China (YZM, NN–MO, TC7, TC5, TC2, BHF6, DH4, FS2, GT20201111, DH1, GXHX211028, TC1, TC3, MSM, TC8, and TC4) and one strain from France (SC01) formed the first cluster, which was distinct from the other clusters (NM2010, USP-BR2017, 90, 14,811, Y98, and ATCC29419). Moreover, the nine strains in this study displayed average ANI values of over 95.0% compared to other Chinese strains.

### Population structure of *M. ovipneumoniae* strains

**Fig. 3:**
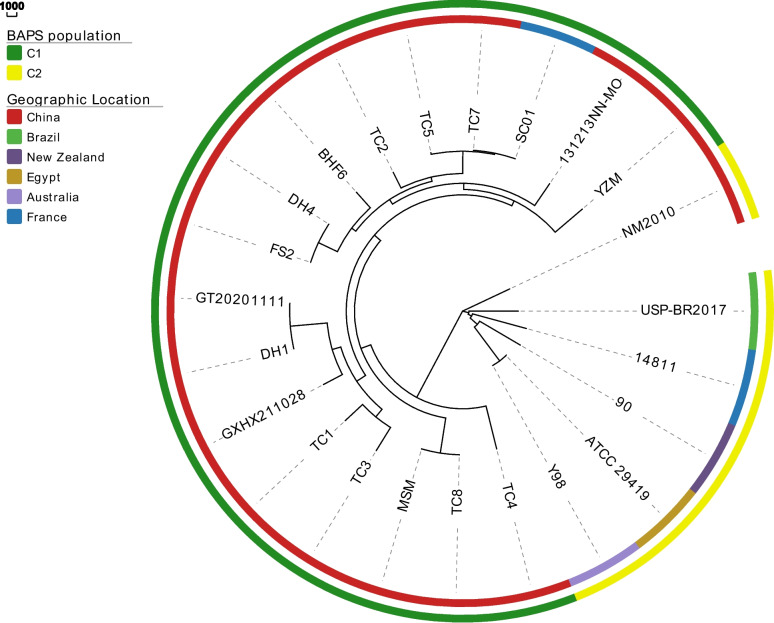
Phylogenetic analysis based on the pangenome of 23 *Mycoplasma ovipneumoniae* strains. Emerald and yellow in outer ring represent the Bayesian analysis of population structure (BAPS) cluster to which each strain is attributed

To study the population structure of *M. ovipneumoniae*, the core genome SNPs of all 23 *M. ovipneumoniae* genomes were analyzed. A total of 219 potential recombination regions were predicted in the core genome of *M. ovipneumoniae* based on the Gubbins analysis (Figure S2). After removing the recombinant regions, core genome SNP alignment was performed to evaluate the population structure, and two distinct clusters were identified (Figure S3). Furthermore, 29,874 non-recombinant SNPs were used to build a recombination-free phylogenetic tree, which revealed a large phylogenetic distance between the two major *M. ovipneumoniae* clusters (Fig. 3). In addition, closely related isolates from China (YZM, NN–MO, TC7, TC5, TC2, BHF6, DH4, FS2, GT20201111, DH1, GXHX211028, TC1, TC3, MSM, TC8, and TC4) and France (SC01) were identified, with comparable degrees of relatedness in the phylogenetic tree (Fig. 3). Moreover, this clade was genetically distinct from foreign strains 90, ATCC 29419, NM2010, 14,811, and Y98. These results are in accordance with the clustering results based on the ANI values, suggesting that the genetic diversity of *M. ovipneumoniae* may be closely related to the geographical distribution of this microorganism.

### Pan-genome analysis of* M. ovipneumoniae* strains

**Fig. 4:**
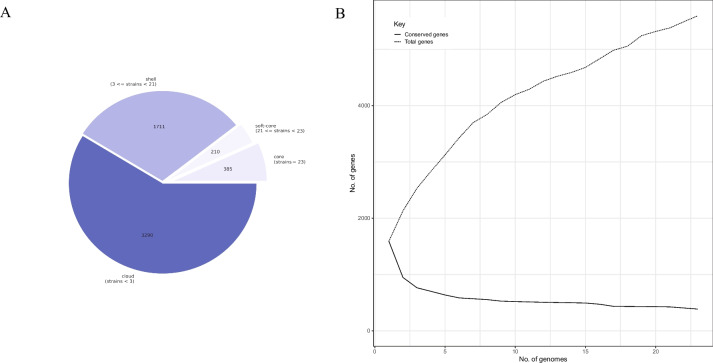
**A** Pie chart showing the distribution of the core genes, soft-core genes, shell genes and cloud genes present in the pangenome of 23 *Mycoplasma ovipneumoniae* strains. **B** Pan-genome and conserved genome. Graph representing the total genes (dotted line) and conserved gene (solid line) of the 23 *M. ovipneumoniae* strains genomes

Pan-genome analysis was performed to explore genetic differences based on the distribution of core microbial genes. The pan-genome of the 23 *M. ovipneumoniae* strains contained 5,596 genes, including 385 core genes, 210 soft core genes, and 5,001 accessory genes (1,711 shell genes and 3,290 cloud genes) (Fig. 4A). The pan-genomic distribution curve revealed that the number of core genes gradually decreased and finally stabilized with an increase in the number of genomes analyzed, whereas the number of total genes showed an increasing trend (Fig. 4B). However, because of the small number of total genomes analyzed, it is difficult to judge whether the pan-genome of *M. ovipneumoniae* is open (i.e., whether the pan-genome has an infinite size) or closed (i.e., whether the pan-genome has a definite size). The distribution of novel and unique genes in *M. ovipneumoniae* was also analyzed. The results showed that the number of novel uncharacteristic genes carried by each strain was between 100 and 200, and more than 2,000 unique genes were detected among the 23 strains (Figure S4).

### Virulence and antibiotic resistance gene analysis

Potential virulence factors in *M. ovipneumoniae* were subsequently analyzed, and ten coding genes (*group_999*, *group_2540*, *group_260*, *group_261*, *group_262, group_264, group_332, group_2053, group_2094,* and *group_2095*) were annotated using the VFDB (Table S2). Both *group_999* and *group_2540* were annotated as cilium adhesins, which mainly contributed to the infection of *M. ovipneumoniae* in goats, while the others were paralog surface adhesins. No antibiotic resistance genes were detected in the pan-genome of *M. ovipneumoniae* when annotated using the CARD database.

### Carbohydrate utilization

**Fig. 5:**
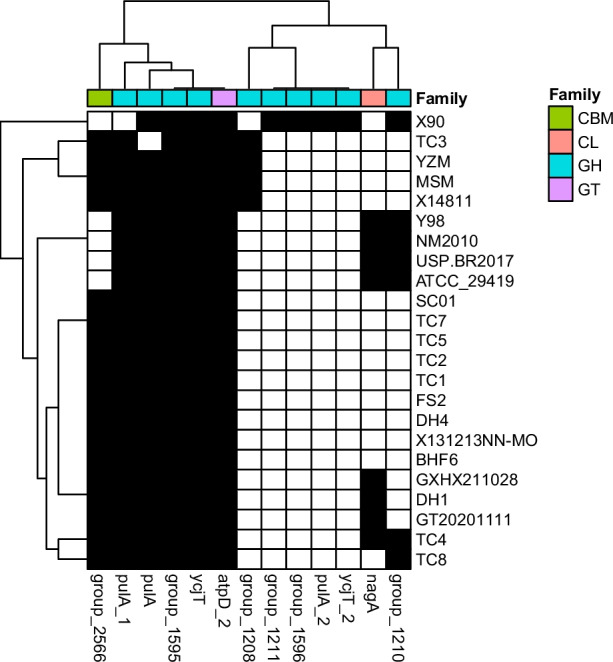
Distribution of carbohydrate-active enzyme genes in 23 *Mycoplasma ovipneumoniae* strains genomes

To analyze the functions related to the carbohydrate metabolism of *M. ovipneumoniae*, genes linked to carbohydrate-active enzymes (CAZymes) were annotated. Four types of enzyme-related genes, glucosidase transferase (GT), glycoside hydrolase (GH), carbohydrate lipase (CL), and the carbohydrate-binding module (CBM), were found in the pan-genome (Fig. 5). All strains carried GH-related (*ycjT* and *group_1595*) and GT-related genes (*atpD_2*), indicating that *M. ovipneumoniae* possesses a wide range of GH activities. In addition, *group_2566*, which contributes to the CBM, was detected in Chinese and French isolates, suggesting that it may be a valuable source of CAZymes. However, *nagA* associated with CL was only found in a few strains (Y98, NM2010, USP.BR2017, ATCC_29419, GXHX211028, DH1, GT20201111, and TC4), demonstrating that *M. ovipneumoniae* strains are generally deficient in CL activity.

## Discussion

The pathogenesis of respiratory diseases and related infectious pathogens is difficult to establish in goats because of interactions between unknown factors and similar anatomopathological patterns. It has been reported that atelectasis, desquamative pneumonia, nodular lymphoid hyperplasia surrounding the bronchioles and vessels, and moderate macrophage exudates are present in only 7 of 15 cases of *Mycoplasma spp* infection. Additionally, 16 of the tested samples contained these lesions and did not show positive results using the microbiological culture technique [21]. Our results are consistent with these findings. Therefore, it is necessary to investigate the features of respiratory diseases and their etiology to identify the causative agents of lung inflammation. We demonstrated that inflammatory cell infiltration and vascular lesions are key clinical symptoms of *M. ovipneumoniae* infection in goats. However, this is insufficient for the diagnosis of *M. ovipneumoniae*-related respiratory diseases.

During the last few decades, WGS-based identification and bioinformatics analysis of clinically pathogenic bacteria have become promising and widely used approaches. Previously, less discriminatory and inefficient techniques such as high-resolution DNA fingerprinting techniques (pulsed-field gel electrophoresis, restriction fragment length polymorphism, and amplified fragment length polymorphism) have been used. In contrast, basic features of the genome and ANI calculations based on WGS data increase the resolution of microbial classification, phylogenetic signals, and other genetic backgrounds derived from comparative genomic analysis [22–24]. We purified nine Chinese isolates, two of which (BHF6 and FS2) exhibited lung consolidation typical of bronchopneumonia. In addition, they had a similar genome size, average GC content, number of open reading frames, and gene organization. The genomes of these nine strains displayed high similarity to the reference genome of *M. ovipneumoniae,* indicating that they were *M. ovipneumoniae* isolates. Phylogenetic trees supported these results and suggested that SC01 may have originated from China.

To understand the physiology of *M. ovipneumoniae* isolates and uncover the minimal genes required for survival, we performed WGS using pan-genomic analysis, which exhibited strong dependability and discriminating capacity, and was appropriate for epidemiological data [25, 26]. Although the core genes of several isolates have been identified using large-scale mutagenesis approaches, systematic evidence of their genomic background, genetic evolution, and biological functions remains scarce. The identification of core genes in uncharacterized microorganisms often relies on sequence alignment and homology mapping of annotated core genes in the reference genomes [27]. In our study, we identified 385 core and 210 soft-core genes (genes missing in up to 5% of all genomes). As the core genes are responsible for the major biological functions of *M. ovipneumoniae*, distinguishing between the core and soft-core genes involved in pathogenic mechanisms and metabolism is promising for exploiting specific medicines or other biological methods to treat diseases associated with *M. ovipneumoniae* infection.

To date, studies on the virulence factors and induced pathogenicity of *M. ovipneumoniae* have mainly focused on *glpF*, *glpK*, *glpD*, *hlyA,* and *hlyC* [28]. However, most of these genes were found in the pan-genome but were not annotated in the VFDB. VFDB annotation identified only cilium adhesin and its paralog, surface adhesin, in *M. ovipneumoniae* (Table S2). Usually, cilium adhesin is present on the cell surface of *Mycoplasma spp.* suggesting a critical role in adhesion. *Mycoplasma pneumoniae* colonization is achieved through the interaction between its expressed adhesin proteins and sulfated glycolipids or sialoglycoprotein molecules in the host respiratory epithelium, causing community-acquired pneumonia [29, 30]. In *M. pneumoniae*, the differentiated terminal organelle was commonly observed to be pointed at and closely related to, the host cell surface; hence, this structure was commonly designated as an “attachment organelle.” Although there are many differences between the structures of *M. pneumoniae* and *M. ovipneumoniae*, *M. ovipneumoniae* adhesion to goat respiratory epithelial cells plays a positive role in the progression of pneumonia. Thus, studies on the microbial mechanisms of pathogenesis are needed to characterize adhesion-mediating molecules [31]. Considering the limited annotation of virulence factors in *M. ovipneumoniae*, it is necessary to characterize other unknown virulence genes and determine their true prevalence and mechanism of action using transcriptomic and metabolomic methods in future investigations.

Owing to the lack of CAZymes, herbivores rely heavily on microbially encoded enzymes to break down cellulose and obtain energy from plant biomass. Although the mechanism of action of CAZymes in *M. ovipneumoniae* has rarely been explored, CAZymes strongly contribute to the metabolism of all sugars in nature, exhibit high selectivity, and display catalytic promise in biochemically complex environments [32]. Herein, we highlight how CAZymes act on the hydrolytic degradation, creation, modification, and rearrangement of glycosidic linkages. Generally, CBM binds to glycans and polysaccharide lyase (PL) enhances the non-hydrolytic cleavage capacity for glycosidic bonds when GT catalyzes the biosynthesis of complex carbohydrate molecules from activated sugars [33, 34]. In addition, most CAZymes establish these reactions with strong specificity for structurally diverse sets of donor substrates. In contrast, acceptors have weaker specificity (monosaccharides, oligosaccharides, peptides, DNA, or lipids) and their binding subsites contribute to the glycosidic bond type in the products [35, 36]. For example, the one linkage/one enzyme concept in the glycan biosynthesis pathway demonstrates that GT facilitates the biosynthesis of glycosidic bonds with high regio- and stereoselectivity [32]. In our study, the hydrolytic degradation of glycosidic linkages in *M. ovipneumoniae* was mostly catalyzed by *ycjT* and *group_1595*, whereas the synthesis of glycosidic bonds was mostly catalyzed by *atpD_2*. In addition, the Chinese isolates carrying *group_2566* showed a stronger ability to recognize and bind glycans. Carbohydrates for uptake and metabolism play a significant role in the production of energy and proteins for a constant supply of variable-surface membrane lipoproteins. Disorders in carbohydrate metabolism promote the escape of *Mycoplasma spp.* from the host humoral defense system, transmission, and proliferation in a variety of locations within the host [37].

## Conclusion

Recent studies have suggested that *M. ovipneumoniae* is a major cause of pneumonia in goats and sheep. In the present study, we characterized the pathogenicity of *M. ovipneumoniae* in goats. We also characterized the genetic diversity of *M. ovipneumoniae* and obtained the core genes and related functional annotations for this species. In future studies, we will investigate molecular detection technology, pathogenic mechanisms, and specific drug applications of *M. ovipneumoniae*.

## Materials and methods

### Species isolation and identification

Epidemiologic investigations were conducted on goat farms throughout Guangxi between 2013 and 2021. Nasal swabs were collected from live goats whose respiratory health status was determined by physical examination of vital parameters and respiratory tract signs.Culture-based assays of pathogenic microorganisms were performed using MacConkey agar plates (Oxoid Ltd., Basingstoke, United Kingdom), blood agar plates (Oxoid Ltd., Basingstoke, United Kingdom), and chocolate agar plates (Difco, BD, Le Pont de Claix, France), according to the manufacturer's instructions. Further identification of individual colonies was performed via PCR and Sanger sequencing using universal 16S rRNA gene primers (27F/1492R:5'-AGAGTTTGATCMTGGCTCAG-3'/5'-CGGTTACCTTGTTACGACTT-3').

Through opening the thoracic and abdominal cavities, lung tissue (*n* = 3, Nanning Shitang Sheep Station, Mashan Lidang Sheep Station and Yizhou Sheep Station) were obtained from three goats that died from respiratory diseases. This experiment was conducted in accordance with the ethical principles of experimental animal welfare of the Committee on Experimental Animal Ethics of the Institute of Veterinary Guangxi Zhuang Autonomous Region. After lung tissue (less than 0.5 cm thick) was fixed in pre-chilled 4% paraformaldehyde (pH 7.2) at 4 ℃ for 24 h, the paraffin sections were prepared and stained with hematoxylin and eosin. The pathological morphology of goat lungs was observed using a Nikon ECLIPSE E200 microscope (Nikon Corp., Tokyo, Japan).

Subsequently, the above lung tissue and nasal swabs (*n* = 6, Beihai Baishan Sheep Station, Dahua Baidan Sheep Station, Fusui Guangyang Sheep Station, Gaotian Sheep Station and Hengxian Sheep Station) were applied for further research. To cultivate and isolate *Mycoplasma spp.*, 0.45-µM filtered material was inoculated onto SP-4 agar plates and liquid SP-4 media at 37 °C for 15 days in aerobiosis. Colonies resembling fried eggs, fermented glucose, or hydrolyzed arginine were identified as the genus *Mycoplasma*. PCR was performed to confirm the presence of *M. ovipneumoniae.* Two fragments were amplified by PCR assay using M1A1:5ʹ-CGAAACTCCCGTGGATGCTA-3ʹ/5ʹ-TTCAACAATTTGCGGATTAA-3ʹ, and M1B1:5ʹ-CGGAGCCATAAAGTTGTAAT-3ʹ/5ʹ-CGAAACTCCCGTGGATGCTA-3ʹ as specific primers. For *M. ovipneumoniae* identification, Sanger sequencing and sequence alignments were performed to identify *M. ovipneumoniae*.

### Genomic DNA extraction and WGS

DNA was extracted and purified from overnight cultures (MRS broth) of *M. ovipneumoniae* using a GeneJET Genomic DNA Purification Kit (Thermo Fisher Scientific, Cleveland, OH, USA) according to standard methods. And genomic DNA was transferred from the column into sterile ddH_2_O, and stored at − 20 °C.

Before sequencing, DNA was visualized on a 1% agarose gel (w/v), and DNA quantification was performed on the Qubit® Fluorometer 3.0 (Invitrogen, Carlsbad, CA, USA). Illumina libraries were constructed on a Hamilton Microlab STAR platform (Hamilton, Bonaduz, Switzerland), followed by quantification using the Kapa Library Quantification Kit (Illumina, San Diego, CA, USA). WGS was performed using the HiSeq platform (Illumina, San Diego, CA, USA). In the final step, raw sequencing data were downloaded for genome assembly and bioinformatic analyses.

### Genome assembly

Before genome assembly, adapters were excluded using Trimmomatic (version 0.36) with a sliding cutoff of Q15 [38]. Subsequently, complete contigs were assembled de novo using SPAdes (version 3.6.2) [39], followed by quality assessment using the QUAST tool [40]. For *M. ovipneumoniae* identification, pairwise average nucleotide identity (ANI) was calculated using the Pyani tool and visualized in a heatmap using the Heatmaply program [41].

### Population genetic structure and phylogenetic analysis

Before PARSNP analysis, the ATCC 29419 genome was used as a reference. Rapid core-genome alignment and visualization were performed using the Parsnp analytical tool [42]. The Gubbins software was used for putative recombination detection and elimination [43]. In addition, the genetic population structure was inferred using BAPS version 6.0 [44], followed by the establishment of an SNP-based neighbor-joining phylogenetic tree [45].

### Genome properties and pan-genome analysis

Genome properties (open reading frame, location, and function) were determined using the Prokka software (version 1.11) [46]. In addition, the visualization and exploration of pan-genome data were processed based on gff and FASTA files using Roary software with a 90.0% identity threshold [47]. Moreover, virulence factors were screened against the Virulence Factor Database (http://www.mgc.ac.cn/VFs/) [48], but also antibiotic resistance genotypes were predicted using ResFinder (https://cge.cbs.dtu.dk/services/ResFinder/) [49], Comprehensive Antibiotic Resistance Database (https://card.mcmaster.ca/) [50], and ARG-ANNOT databases [51].

### Supplementary Information

**Additional file 1: Figure S1.** (A) M. ovipneumoniae colonies on SP-4 agar plate showing dew drop appearance. (B) M. filiformis colonies on SP-4 agar plate showing fried egg appearance. (C) Microscopic morphology of M. ovipneumoniae. **Figure S2. **Distribution of recombinant sequences over the *Mycoplasma ovipneumoniae* core genome. Black segments below the recombinant regions indicate recombination hotspots. **Figure S3. **Results of genetic structure analysis of strain population. **Figure S4.** New genes and unique genes. Graph representing new genes (solid line) and unique genes (dotted line) of the 23 *Mycoplasma ovipneumoniae* genomes.

**Additional file 2: Table S1.** Genomic statistics of the public genome sequences available in NCBI database. **Table S2.** Annotation results of potential virulence genes.

## Data Availability

The datasets used and/or analysed during the current study are available in the NCBI Sequence Read Archive repository, [SRR11947113, SRR13638375, SRR13624992, SRR13626389, SRR13636541, SRR13616361, SRR13616686, SRR14321785, SRR14000708].

## Abbreviations

VFDB: Virulence Factor Database
MALDI-TOF: Matrix-assisted laser desorption/ionization-time of flight mass spectrometry
LAMP: Loop-mediated isothermal amplification
AST: Antimicrobial susceptibility testing
WGS: Whole genome sequencing
ARGs: Antimicrobial resistance genes
SNPs: Single nucleotide polymorphism
CARD: Comprehensive Antibiotic Resistance Database
CAZymes: Carbohydrate-active enzymes
GT: Glucosidase transferase
GH: Glycoside hydrolase
CL: Carbohydrate lipase
CBM: Carbohydrate-binding module
ANI: Average nucleotide identity
PL: Polysaccharide lyase
