# Supplementary material for: Mesomycoplasma ovipneumoniae from goats with respiratory infection: pathogenic characteristics, population structure, and genomic features
Source: BMC Microbiol. 2023 Aug 14;23:220. doi: 10.1186/s12866-023-02964-0 (PMC10424369; doi:10.1186/s12866-023-02964-0)
Supplement: Supplementary file 1 — Additional file 1: Figure S1. (A) M. ovipneumoniae colonies on SP-4 agar plate showing dew drop appearance. (B) M. filiformis colonies on SP-4 agar plate showing fried egg appearance. (C) Microscopic morphology of M. ovipneumoniae. Figure S2. Distribution of recombinant sequences over the Mycoplasma ovipneumoniae core genome. Black segments below the recombinant regions indicate recombination hotspots. Figure S3. Results of genetic structure analysis of strain population. Figure S4. New genes and unique genes. Graph representing new genes (solid line) and unique genes (dotted line) of the 23 Mycoplasma ovipneumoniae genomes. [file 12866_2023_2964_MOESM1_ESM.pptx]

## Slide 1
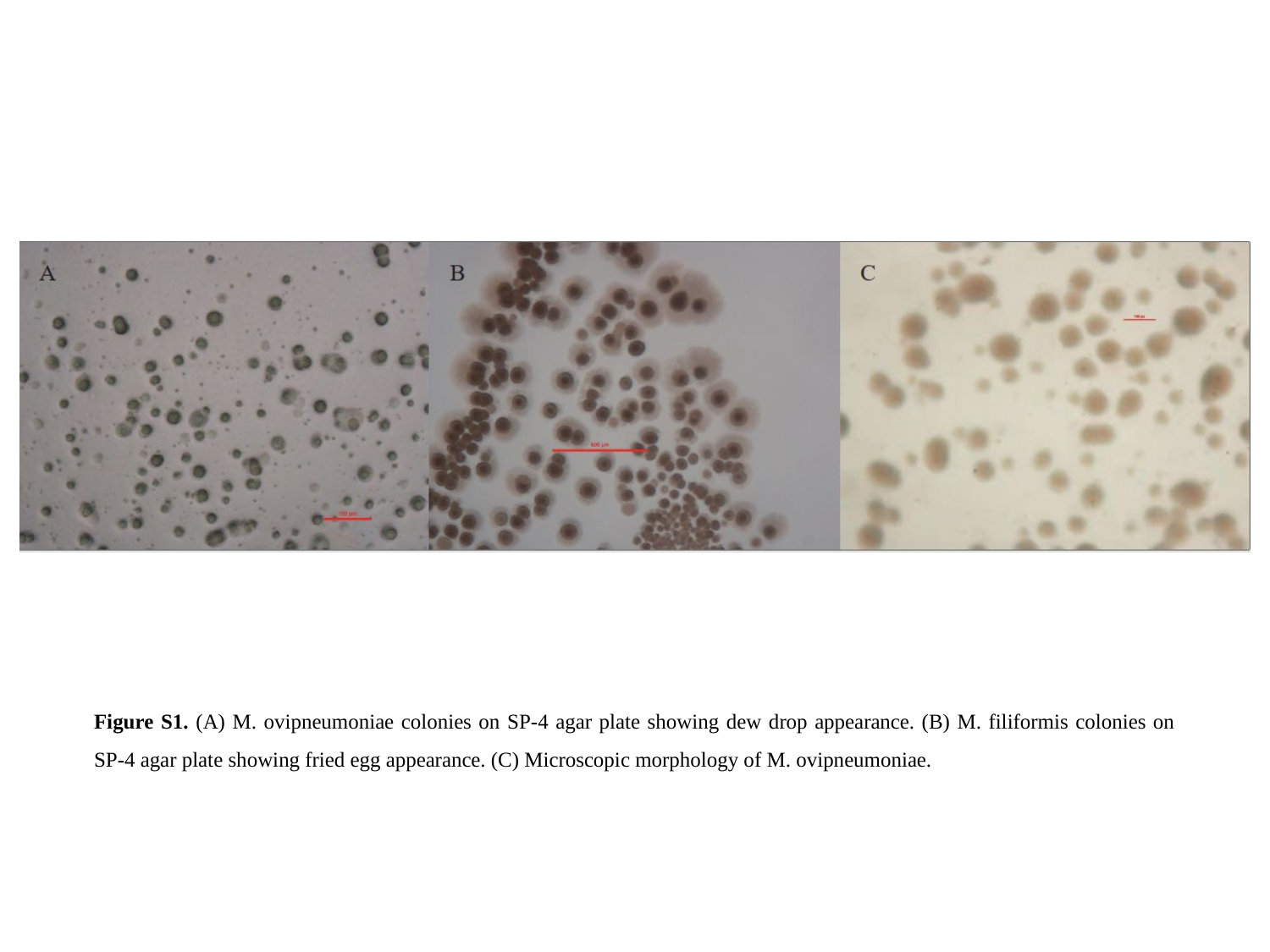

Figure S1. (A) M. ovipneumoniae colonies on SP-4 agar plate showing dew drop appearance. (B) M. filiformis colonies on SP-4 agar plate showing fried egg appearance. (C) Microscopic morphology of M. ovipneumoniae.

## Slide 2
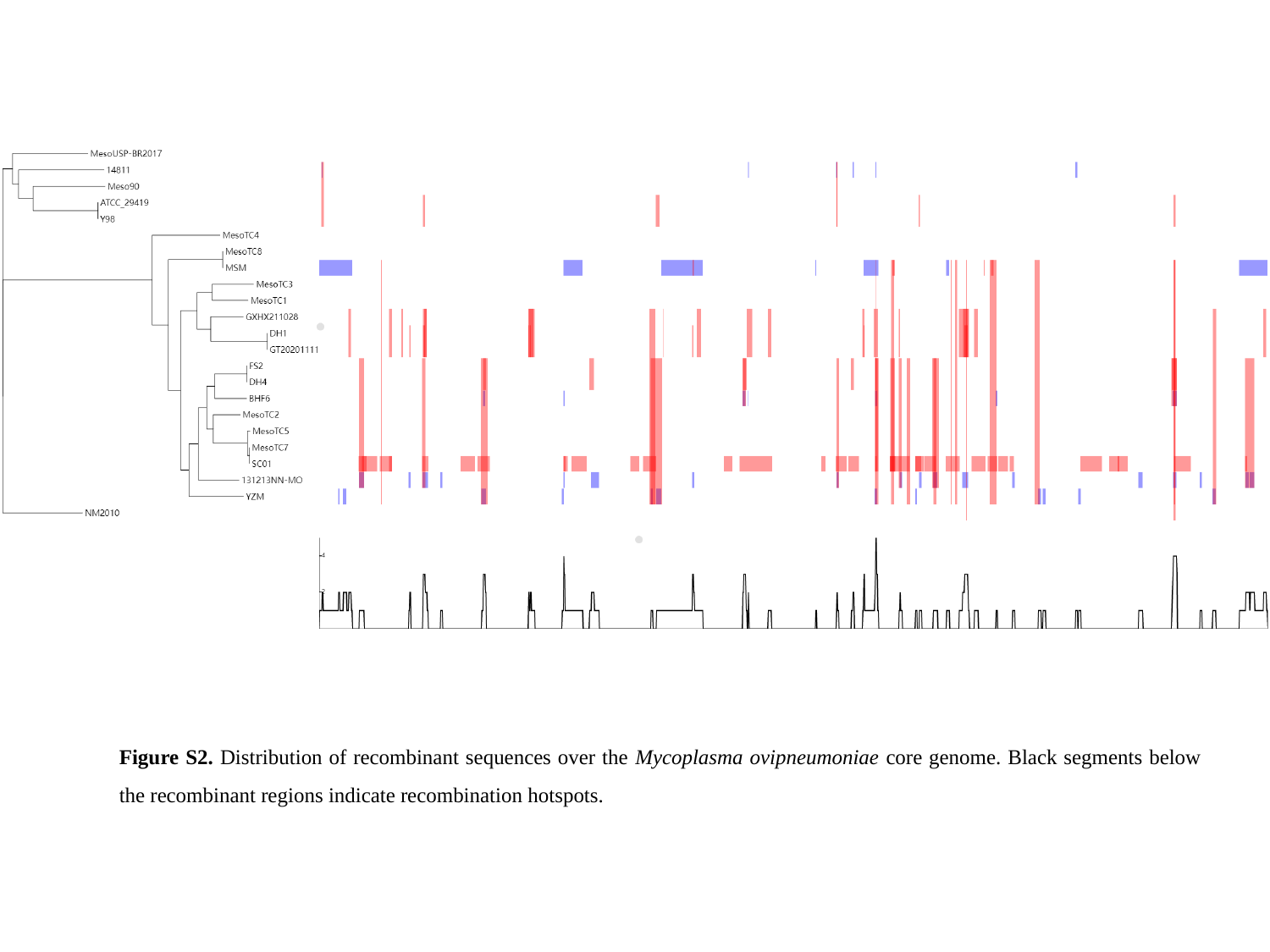

Figure S2. Distribution of recombinant sequences over the Mycoplasma ovipneumoniae core genome. Black segments below the recombinant regions indicate recombination hotspots.

## Slide 3
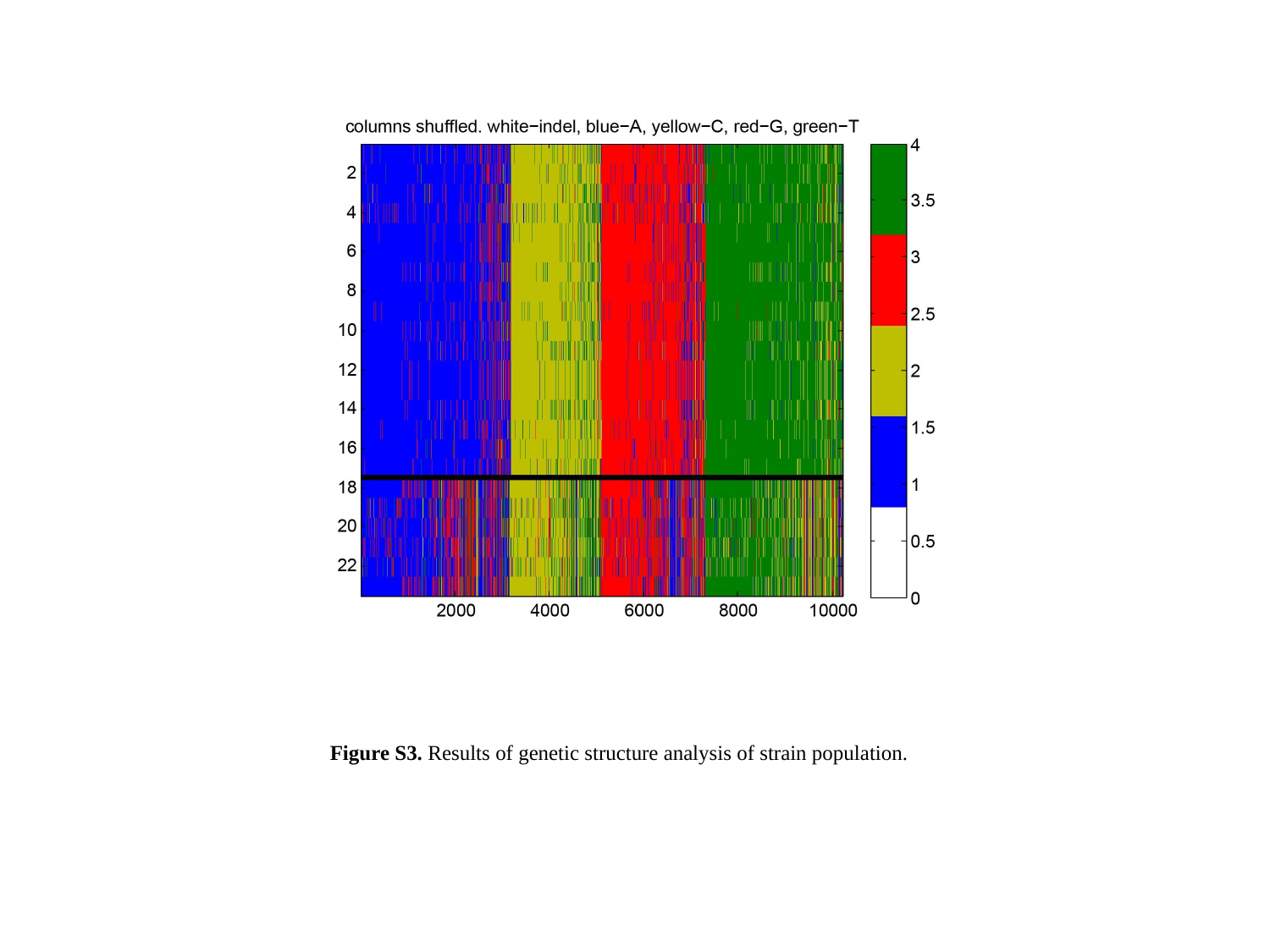

Figure S3. Results of genetic structure analysis of strain population.

## Slide 4
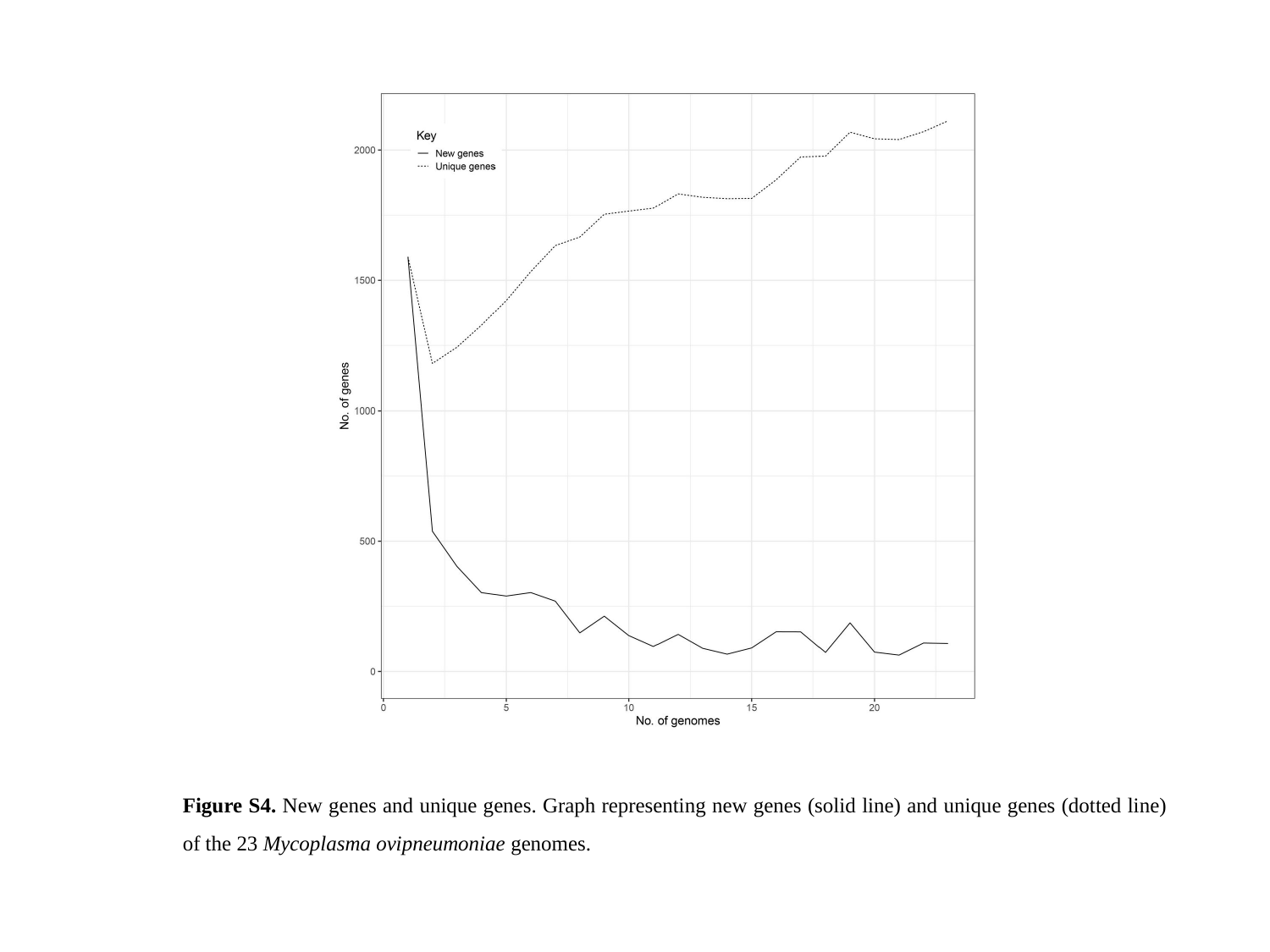

Figure S4. New genes and unique genes. Graph representing new genes (solid line) and unique genes (dotted line) of the 23 Mycoplasma ovipneumoniae genomes.
